# Imported acute viral hepatitis E. case report

**DOI:** 10.1186/1471-2334-13-S1-P73

**Published:** 2013-12-16

**Authors:** Lilia Cojuhari, Victor Pântea, Gheorghe Plăcintă, Valentin Cebotarescu, Valentina Smeşnoi, Paulina Jimbei

**Affiliations:** 1 Department of Infectious Diseases, Faculty for Continuing Medical Education, Nicolae Testemițanu State Medical and Pharmacy University, Chişinău, Republic of Moldova; 2 Toma Ciorbă Clinical Hospital for Infectious Diseases, Chişinău, Republic of Moldova; 3 Chiril Draganiuc Institute of Phthisiopneumology, Chişinău, Republic of Moldova

## Background

Viral hepatitis E is an infectious disease which is mainly spread in Asia (India, Pakistan) and in some regions of Africa, Central and South America, but there were described some imported cases in Spain and Italy. Hepatitis E causes significant morbidity and mortality in young adults, particularly among pregnant women and patients with pre-existing chronic liver disease.

## Case report

We present the case of a 54 year-old female patient, which was admitted to the Clinical Hospital for Infectious Diseases “Toma Ciorbă” with 38.5°C temperature, with epigastric and right hypochondriac pain, abdominal discomfort, and jaundice. The clinical exam showed abdominal tenderness on palpation in the epigastric region and in the right hypocondrium, the liver being 2-3 cm below the right costal margin, and the spleen 1-2 cm. Before the admission, the onset of the symptoms was sudden with up to 40°C temperature, pain in the right hypochondrium, permanent nausea, fatigability, decreased appetite and dark urine. Laboratory examination showed: total bilirubin 31.0 µmol/L; direct - 7.6; ALAT - 1500 U/L; ASAT- 1300 U/L; alkaline phosphatase- 726 U/L; GGTP- 279 U/L; prothrombin index - 56%. Personal records: in 2007 the diagnosis of chronic viral hepatitis C was established; bilirubin, ASAT and thymol test with normal values. Markers of viral hepatitis: total anti-HCV positive; total anti-HBc positive; anti-HAV IgM negative; anti-HBc IgM negative; anti-HCV IgM positive; anti HDV IgM negative; total anti HDV negative; anti-CMV IgM negative; anti-CMV IgG positive; anti-EBV-VCA IgM- negative; anti EBV-VCA IgG positive; anti-HBs negative; anti-HEV IgM O3- positive; anti HEV IgG O3- positive. From the epidemiological history it was established that five years before she had travelled to Bulgaria. Instrumental investigations: ultrasonographic examination - the liver, right lobe 15.5 cm; the left lobe 6.0 cm; the spleen 12.3x5.0 cm; portal vein 1.0 cm; splenic vein 0.7 cm; FibroScan: the average coefficient of hepatic elasticity 6.8 kPa, which corresponds to F0-F1 stage of hepatic fibrosis (according to the Metavir scale).

Symptomatic and pathogenetic administered treatment resulted in clinical and biochemical improvement. The patient was monitored clinically and biochemically at 1 and 2 months. Cytolysis syndrome tests had normal values.

## Conclusion

Clinical symptomatology was characterized as an acute viral hepatitis, serologically confirmed, revealed by anti HEV IgM O3 and anti-HEV IgG O3, the evolution being benign with a general satisfying state and normal values of cytolysis syndrome indices.

